# Nanofabrication of Isoporous Membranes for Cell Fractionation

**DOI:** 10.1038/s41598-020-62937-5

**Published:** 2020-04-09

**Authors:** Ainur Sabirova, Florencio Pisig, Naganand Rayapuram, Heribert Hirt, Suzana P. Nunes

**Affiliations:** 1 King Abdullah University of Science and Technology (KAUST), Biological and Environmental Science and Engineering (BESE) Division, Advanced Membranes and Porous Materials Center, 23955-6900 Thuwal, Saudi Arabia; 2 King Abdullah University of Science and Technology (KAUST), Nanofabrication Core Laboratory, 23955-6900 Thuwal, Saudi Arabia; 3 King Abdullah University of Science and Technology (KAUST), Biological and Environmental Science and Engineering (BESE) Division, Center for Desert Agriculture, 23955-6900 Thuwal, Saudi Arabia

**Keywords:** Plant sciences, Nanopores

## Abstract

Cell fractionations and other biological separations frequently require several steps. They could be much more effectively done by filtration, if isoporous membranes would be available with high pore density, and sharp pore size distribution in the micro- and nanoscale. We propose a combination of two scalable methods, photolithography and dry reactive ion etching, to fabricate a series of polyester membranes with isopores of size 0.7 to 50 μm and high pore density with a demonstrated total area of 38.5 cm^2^. The membranes have pore sizes in the micro- and submicro-range, and pore density 10-fold higher than track-etched analogues, which are the only commercially available isoporous polymeric films. Permeances of 220,000 L m^−2^ h^−1^bar^−1^ were measured with pore size 787 nm. The method does not require organic solvents and can be applied to many homopolymeric materials. The pore reduction from 2 to 0.7 μm was obtained by adding a step of chemical vapor deposition. The isoporous system was successfully demonstrated for the organelle fractionation of Arabidopsis homogenates and could be potentially extended to other biological fractionations.

## Introduction

Different biomedical and diagnostic technologies, such as cancer cell separation, biosensing, controlled drug delivery, microfluidic devices for organ-in-a-chip, as well as investigations in plant and bioscience relay on effective separation processes. The main hurdle for an effective separation by filtration is the limited availability of highly uniform pores in the size of tens to a few micrometers, a range relevant for these applications. The majority of commercial membranes, prepared in large-scale by a classical solution casting and non-solvent induced phase separation (NIPS), have a broad pore size distribution, limiting their application when strict selectivity is essential. There is only one kind of commercial polymeric isoporous membranes in the market: the track-etched membranes (e. g. Nuclepore, Cyclopore), prepared by the bombardment of fission fragments in a nuclear reactor or ion beams in high energy accelerators, mostly available with pores of a few micrometers or down tenths of micrometer^1,2^. The track-etched membranes are usually made of polycarbonate (PC) or poly(ethylene terephthalate) (PET). Their pore density is low, they are normally commercialized in small discs, the isoporosity is not always perfect due to the possible overlap of tracks during the bombardment preparation step^3^. Beside track-etching, among the most interesting research approaches to develop isoporous membranes in the last decade is the self-assembly and non-solvent induced phase separation (SNIPS) of block copolymers, also a relevant topic in our group^4^. Most membranes under this category have pores in the range of 20–60 nm, with some reports down to a few nanometers. While the SNIPS process can be done in large membrane machines, the obtained morphology is highly dependent on the composition of the block copolymer, the solvent and conditions used for the casting process. The material itself is costly and the optimization to provide membranes without any defect to fulfil the expectations of the biotech industry is challenging. Few other approaches proposed for the isoporous membrane preparation and explored in a lab scale are listed in Table 1. Breath figures^5–7^ have been used by different groups to promote the formation of regular pores in the microfiltration range. The pores are obtained by spreading polymeric solutions at the interface between humid air and a cold surface. The solvent evaporation contributes to the temperature decrease, and favors the condensation of water droplets, which work as nuclei for the pore generation. The pore size and distribution are highly dependent on the solvent, normally apolar, polymer concentration and solubility, surfactant additives and the humidity level. Another reported approach is the incorporation of sacrificial silica particles in polymer films, following a combination of micromolding, float-casting and exposure to hydrofluoric acid^8^. A complex combination of molecular templating and supramolecular assembly of discotic components, followed by the template removal by treatment with sodium hydroxide has been recently reported^9^. A breakthrough in applications such as cell fractionation and biomolecules purification would be primarily achieved if better isoporous membranes would be available^10^. Besides the pore size uniformity, an optimum membrane is expected to have a high porosity for high flux, mechanical stability and preferentially flexibility for an easy handling and integration into a device. The manufacturing process should be scalable, especially if preparative fractionations are targeted. If the application involves the exposure to organic solvents, a polymeric material with high chemical stability is necessary. Having a fabrication process that can be easily applied to different polymeric materials is therefore highly advantageous. Nanofabrication methods are constantly progressing. Other lithographic methods have been proposed for the membrane preparation before, but not with the sufficient holistic combination of large filtration area, small pore size and high pore density, requested for cell and protein separations in real applications.

**Table 1:** Non-litographic methods of isoporous membrane preparation.

Method	Materials	Pore Diameter (μm)	Comments	Ref.
Track-etching	PET, PC	0.2–2	Tracking by high energy particles in a nuclear reactor or accelerators followed by etching in caustic medium; regular size pores; pore merging might occur, low pore density; no solvent required; commercialized in discs with 47 mm (or lower) diameter	^1,2^
Anopore/Anodisc	Anodic Aluminum Oxide (AAO)	0.02–0.2	Anodization of aluminum by an electrochemical process; commercialized in discs with 47 mm (or lower) diameter; inorganic; highly brittle	^27^
Block copolymer self-assembly, followed by selective etching	Copolymers with polylactide blocks	0.02–0.06	Self-assembly in selective solvent, followed by long etching of the polylactide blocks in NaOH; lab-scale demonstration	^28^
Block copolymer SNIPS	Block copolymers	0.002–0.1	Self-assembly in selective solvents and macrophase separation in water; asymmetric; under consideration for commercialization (start-up Terapore), mostly with polystyrene and vinyl pyridine blocks;	^4^
Breath figures	Block copolymers/ homopolymers	1–5	Water droplets condensation on spread polymer solution (preferentially apolar solvents); highly dependent on humidity level and other conditions; lab scale demonstration	^5–7^
Incorporation of sacrificial particles	Crosslinked acrylate	0.03–0.3	Float-cast microsieves prepared by photocrosslinked acylate monomers in solution with silica microspheres, followed by etching with hydrofluoric acid; lab scale demonstration	^8^
Molecular templating/supramolecular assembly	Epoxy/ linoleic acid	0.001–0.002	Crosslinking of systems with assembled discoid templates in solution, followed by etching; lab scale demonstration	^9^

Pioneering reports in the field are listed and compared in Table 2. The first use of lithography for the fabrication of microporous systems was reported by Ogura *et al*.^11^ for inorganic membrane and by Brauker *et al*.^12^ for organic membrane. Brauker’s team could fabricate 1 cm^2^ of a polyimide porous film with 20-μm pores and 5 μm interpore distances for the purpose of implanting living cells. Parylene, a poly(p-xylene) synthesized by chemical vapor deposition, was initially used for the protection of electronic printed circuits. It was later considered for the fabrication of porous structures by photolithography processes, following a procedure completely different than that used in our work. The strategy consisted in patterning a preformed dense filme of Parylene to give rise to the pores^13–17^. Only a couple of other polymers have been reportedly used for membrane fabrication by lithography. Among the few examples are epoxy-based photoresists (e. g. SU-8)^18,19^, also frequently applied in the electronic industry for other purposes and polydimethylsiloxane (PDMS)^20–22^. Huh *et al*.^20^ first demonstrated the fabrication of controllable PDMS microporous membrane having better porosity along with larger active area (1 × 2 cm) than previous reports, effective for the chosen application, but still smaller than what would be needed for most filtration purposes. By using soft lithography and wet etching techniques, they successfully fabricated membranes (with 10 µm or 7 µm pores), which served as cell culturing support for a microfluidic device, mimicking human lung functions^20^. The microfabrication protocol for the reported PDMS membrane, as well as for other examples in Table 2, involves several complex phases with critical steps making it a challenging process, at least in part, with overall fabrication time of ≈ 33 hours or higher^23^. The soft lithography technique in general requires expensive and fragile patterned silicon master wafers for microelectromechanical systems (MEMS). When considering other applications, PDMS is frequently used as dense film for gas and vapor separations. However, for separations under pressure, requiring membranes with well-defined pores, PDMS is not the best choice, since it is a soft rubbery material and it easily compacts with pore deformation. PDMS also absorbs drugs and biological molecules, potentially affecting the effectiveness of screening studies on a targeted drug supply or cell-signaling assay^24^. For ultrafiltration membranes, glassy or semicrystalline polymers are better materials than rubbery ones to guarantee the dimension stability of the pores. Different filtration applications have different requirements and priorities (e. g. cost, stability), driving the choice of the material to be used. Therefore, a membrane manufacturing process applicable to a broad range of polymeric materials is highly advantageous. For biomedical devices using porous layers, the choice of materials is again of primordial importance, as well as the thickness and pore distribution layout^25,26^. For *in vitro* cells culturing platforms, porous membranes are used as a barrier either between two different types of cells or between cells and the drug media. The membrane thickness and surface wettability are important. Potential unspecific adsorption should be avoided. Flexibility in the membrane material used in different applications can be very convenient.

**Table 2:** Membranes fabricated with lithography assistance.

References and authors	Material	Pore size (μm)	Interpore space (μm)	Max. Area (cm^2^)	Manufacturing methodology
***Inorganic membranes***					
Ogura *et al*.^11^	Silicon Nitride	1–1.8	N/A	N/A	Si_3_N_4_ deposition, electron beam lithography, plasma etching
Vaeth^29^	Silicon	3–5	40	1.62	Deposition, hard mask lithography, fluid channel lithography, etching
Warkiani *et al*.^30^	Metal	2.5 × 8	N/A	78.5	Multilevel UV-lithography, electroplating
Carter *et al*.^31^	Glass (SiO_2_)	0.5, 3	N/A	14	SiO_2_ deposition, photolithography, RI etching
Salminen *et al*.^32^	Silicon Nitride	3	N/A	-	Lithographic laser writing, RI etching
***Organic membranes***					
Brauker *et al*.^12^	Polyimide	20	2–5	1	Polyimide nonporous film formation, lithography, oxygen curing
Zheng *et al*.^13^	Parylene	10	10	0.36	Dense Parylene film formation, O_2_ plasma, etching
Huh *et al*.^20^	PDMS	10	30	2	Soft lithography, wet etching
Hosokawa *et al*.^33^	PET	4–2	60	4	Photolithography-based electroforming
Xu *et al*.^14^	Parylene	6	N/A	0.36	Dense Parylene film formation, Cr/Al deposition, wet etching
Warkiani *et al*.^18^	SU-8 photoresist	3 × 12	2–4	—	UV-lithography, epoxy (SU-8) photopolymerization
Chen *et al*.^21^	PDMS	4–20	8–200	9	O_2_ plasma, photolithography, dry RI etching
Harouaka *et al*.^15^	Parylene	4–7	N/A	0.5	Dense Parylene film formation, photolithography, etching
Kim *et al*.^16^	Parylene	0.8–4	N/A	0.42	Dense Parylene film and Ti mask formation, photolithography, dry etching
Tang *et al*.^34^	PEGDA	5.5–8	24	0.81	Photolithography, molding
Zhou *et al*.^17^	Parylene	8	12	1	Dense Parylene film formation, lithography, aluminum etching
Adams *et al*.^19^	SU-8 photoresist	5–9	20	<0.64	Dense epoxy-based film formation, photolithography
Musah *et al*.^22^	PDMS	7	30	N/A	Soft lithography, wet etching
This work	**Mylar**_,_ **Kapton**	**0.7**–**50**	**2**–**50**	**38.5**	**Mylar and Kapton dense films patterned by photolithography and dry RI etching. For submicron pores, a CVD step was added**.

**Figure 1:**
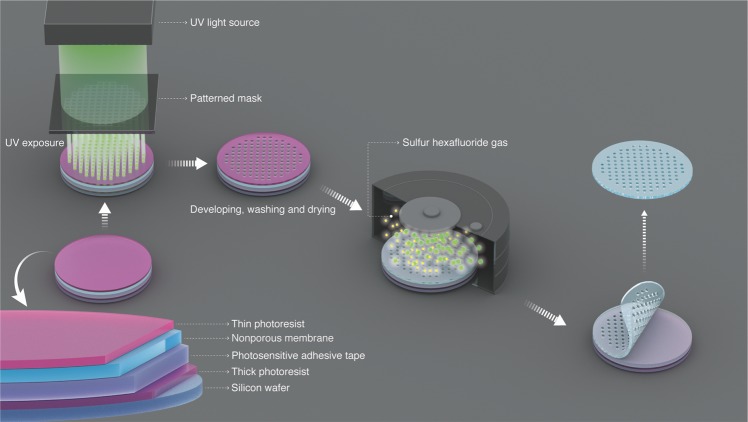
Schematic illustration of the isoporous polymer membrane fabrication method. Combination of photolithography and dry reactive ion etching.

We propose in this work a new method of isoporous polymeric membrane fabrication, applicable to a large portfolio of commercial homopolymers. We demonstrated the preparation of membranes constituted by Mylar and Kapton, materials known for their high chemical, mechanical and dimentional stabilities. The membranes have a filtration area of 38.5 cm^2^, pore size between 0.7 and 50 µm, with high pore density. The method combines photolithography and dry reactive ion etching, as depicted in Fig. 1, providing an exact pore tuning down to 1 µm. To further reduce the pore size to the submicron range, we added chemical vapor deposition (CVD). The three methods are scalable and are individually applied under different conditions and parameters in the industry for the production of electronic devices. Here their combination provides a unique technology for the preparation of membranes with unique pore characteristics, without the solvent and water waste characteristic of the NIPS method. We applied the membranes for the mild fractionation of organelles in plant systems in substitution of more tedious centrifugation steps.

## Results and Discussion

### Membrane fabrication and characterization

**Figure 2:**
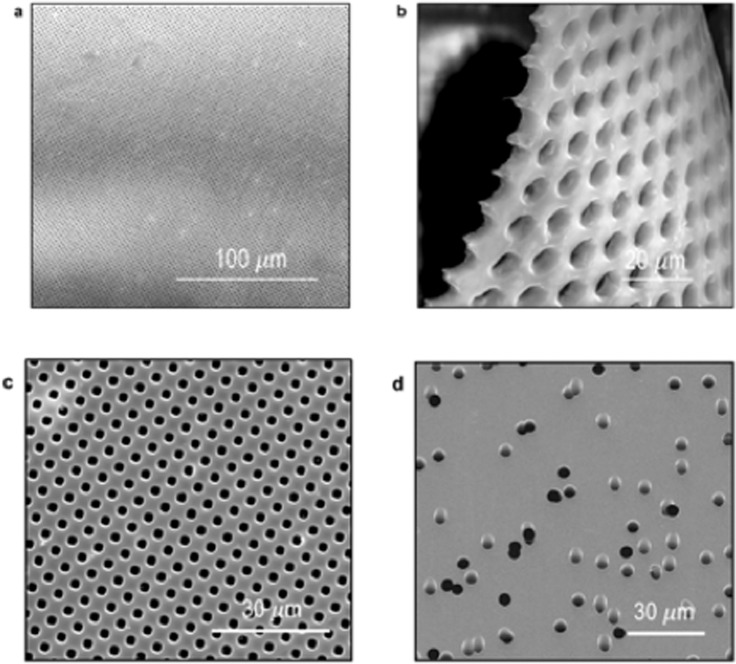
Nanofabricated isoporous membranes. SEM images of (**a**–**c**) Mylar membranes prepared by photolithography/dry reactive ion etching in this work and (**d**) a commercial track-etched membrane.

**Figure 3:**
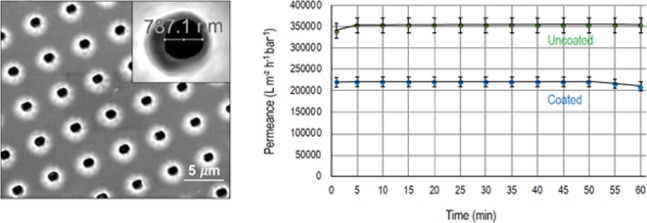
CVD-coated Mylar membranes. (**a**) SEM image with inset showing a high magnification of a pore, after CVD deposition of Parylene (diameter 787 nm). (**b**) Water permeation of Mylar porous membranes fabricated with 2 μm pores before and after CVD deposition.

We chose Mylar, a biaxially-oriented poly(ethylene terephthalate) film, as the first base material for the membrane fabrication. The polymer provides the mechanical flexibility and strength for an easy integration into Millipore Amicon filtration cells for the performance characterization, and later the integration in modules for diverse applications requiring higher pressure. The first step was the photomask preparation, as shown in Fig. 1. Photoresist-chromium-glass masks with 3-inches diameter were designed and accomplished with up to 250 million pores of 2 µm diameter. The interpore distance of 2 µm guaranteed a very high pore density. Masks were also obtained with 5, 10, 25 and 50 µm pore sizes. The masks can be reused innumerous times for the further membrane preparation with a variety of materials. The masks were used to expose 2.5-µm-thick polyester films to UV in a photolithographic set-up. After the UV exposure, the films were submitted to a dry reactive ion etching process. The scanning electron microscopy (SEM) images of the obtained membranes with highly ordered pores and high pore density can be seen in Fig. 2. The comparison between our nanofabricated Mylar membrane and a commercial track-etched PC membrane is shown in Fig. 2d. Figure 2c reflects a membrane pore size of 2 µm, a tight distribution, with interpore spaces of 2 µm, corresponding to a density of 4.13 × 10^6^ pores per cm^2^. This pore density is at least one order of magnitude higher than that of track-etched membranes with similar pore size, depicted in Fig. 2d. The water permeation measurement was performed using a stainless-steel filtration cell with an active membrane area of 1 cm^2^, an initial feed (water) volume of 300 mL and a trans-membrane pressure of 0.03 bar. A stable water permeation of 352,000 L m^−2^ h^−1^ bar^−1^ was measured, as shown in Fig. 3, and is much higher than the 276,000 L m^−2^ h^−1^ bar^−1^ permeation of track-etched membrane with the similar 2 µm pores size, reported by STERLITECH Corporation.

With the equipment used in this work, a radius of 1 µm was the minimum we could obtained with acceptable mechanical stability and pore regularity.

Besides Mylar, we successfully fabricated isoporous membranes using Kapton, a polyimide stable at temperatures up to 400 °C. The fabrication protocol was practically the same, except for increasing the etching time, as the thickness of Kapton used film was 7.5 µm. Fig. S1 shows SEM images of Kapton membranes prepared with 2 µm and 10 µm pores sizes.

To bring the pore size down to the submicron-range, an additional step was necessary. We applied another relatively simple technology also used in the electronic industry: controlled chemical vapor deposition (CVD). We promoted the partial deposition of Parylene C on the surface of the membranes with 2 µm pores. The deposition reduced the pore diameters to 787 nm, as shown in Fig. 3. The pores become slightly asymmetric afer the Parylene deposition. See the SEM images of the top and bottom of the membranes in Fig. S2. A water permeation of 220,000 L m^−2^ h^−1^bar^−1^ was measured for the modified membranes. Parylene has been used for the first time for this kind of approach. In a few other approaches related to membranes, listed in Table 2, Parylene was used as a dense starting film for lithography.

### Organelle fractionation

The membranes were applied for the fractionation of cell organelles of *Arabidopsis thaliana*. Cell organelles such as chloroplasts, mitochondria, nuclei, peroxisomes etc., carry out the majority of the metabolic processes in plants and other biological organisms. The cell fractionation of different organelles is normally performed by a combination of several biochemical methods. Typically, the first step in the cell fractionation is the preparation of a homogenate by breaking open the cells that contain all the membrane-bound organelles with distinct size, charge and density. The organelles separation in this homogenate is frequently done by ultracentrifugation. The homogenate is spun at high speed to separate the components on the basis of their density. As in the case of many biological separation procedures, cell fractionation is a time-consuming process. The homogenates must be centrifuged for relatively long periods of time, depending on the organelle that is being isolated, the centrifugation is repeated and re-suspended in fresh re-suspension solution compromising the organelle integrity. The use of a series of isoporous membranes with finely tuned pore sizes is expected to facilitated the fractionation leading to an effective separation under mild conditions.

*Arabidopsis thaliana* ecotype Columbia-0 was chosen for this study. *Arabidopsis*, a member of the Brassicaceae family is related to several vegetable crop plants such as broccoli, cabbage, Brussels sprouts, cauliflower etc., and has for long been used as a model organism by plant scientists. This is owing to a number of features including the availability of high-quality genome sequence, a short life cycle, its ability to produce a lot of seeds, amenable for genetic modification as well as several genetic tools for research. Due to its small size, *Arabidopsis* is not an organism for biochemical investigations and therefore, improved techniques in this regard are highly solicited.

**Figure 4:**
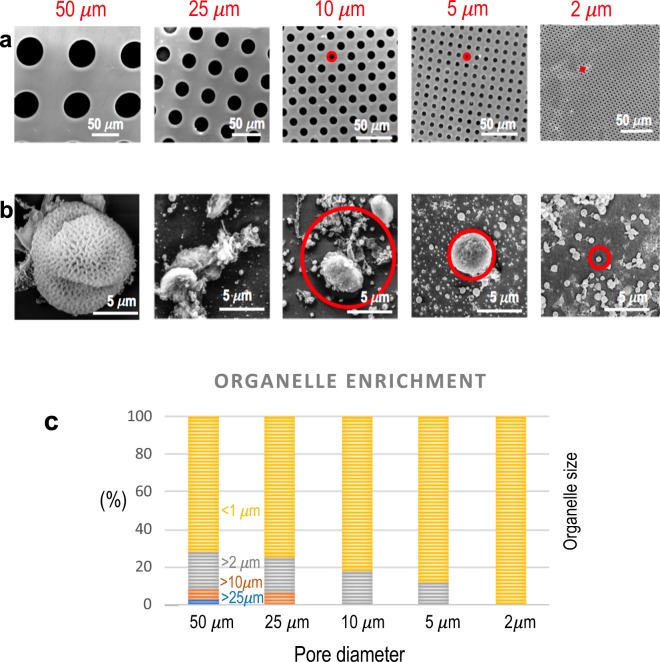
Organelles separation by membrane filtration. (**a**) SEM images of Mylar isoporous membranes fabricated with pore sizes of 2, 5, 10, 25 and 50 μm. (**b**) Organelles of different sizes permeated and fractioned through the membranes imaged above; red circles highlight the pore sizes through which the organelles were collected. (**c**) Statistic distribution of organelles sizes in the permeate of each membrane.

**Figure 5:**
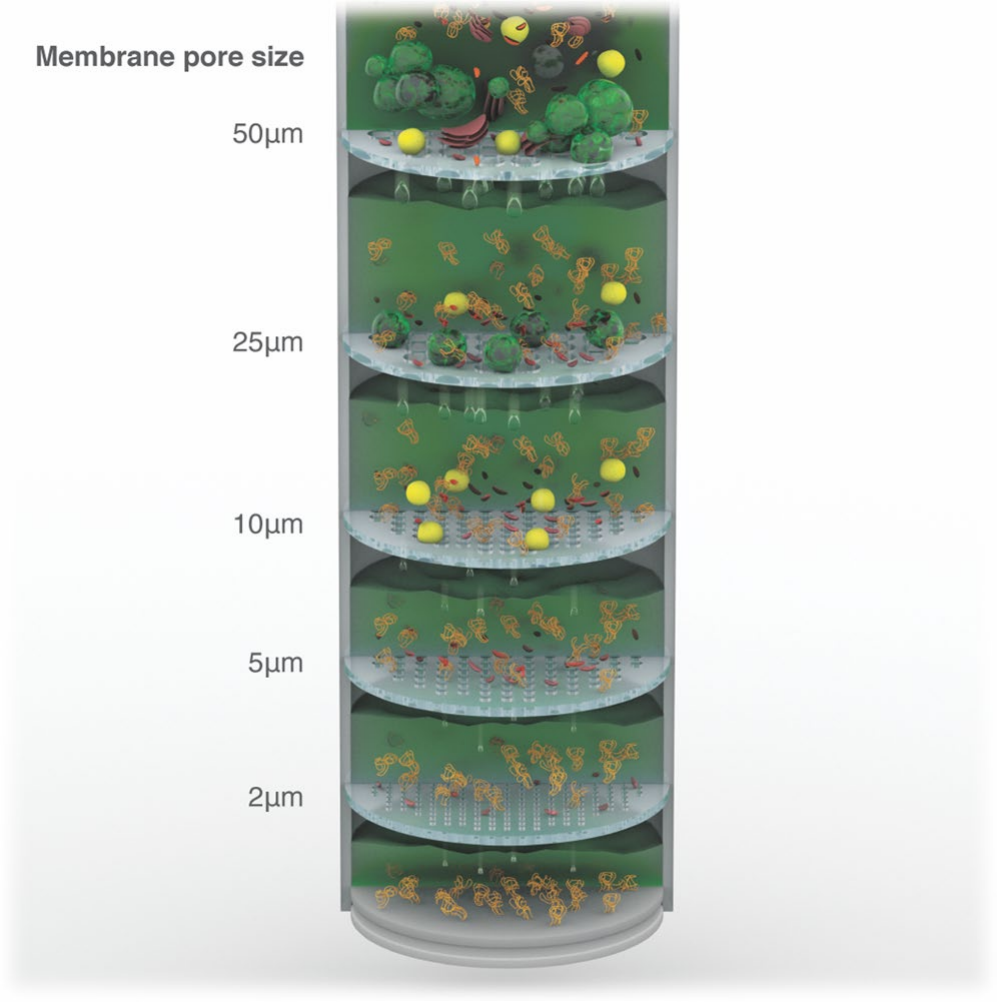
Schematic illustration of plant organelles sorting using five isoporous membranes with different pore sizes.

From SEM images obtained from each fraction, exemplified in Figs. 4 and S3, organelles of various sizes were counted using the ImageJ analysis software. The stacked or clustered bar graph shows organelles of different sizes that were quantified in each of the permeates. For the ease of comparison, the numbers are represented as percentages. The permeate of 50 µm membranes contains a few organelles that are more than 25 µm in size along with structures smaller in size. Similarly, the permeate of membranes with smaller pores contains smaller organelles. The smallest structures are the most abundant in each of the permeates. As expected, the permeate of the membrane with 2 µm pores contains only structures smaller than 1 µm. Each membrane retains organelle aggregates that are larger than the pore size of the corresponding membrane. The separation in Fig. 4 was obtained in experiments with a cascade of single cells with membranes of decreasing pore size (from 50 to 2 µm) **(**Fig. S4**)**. A more elegant arrangement of membranes with different pore sizes is depicted in Fig. 5. A CAD scheme for an integrated set-up is shown in Fig. S5.

## Conclusion

There are only a few existing approaches for the nanofabrication of highly ordered, scalable, polymeric membrane and they have drawbacks, such as being complex and time-consuming methodologies, limited to only few applicable polymers, as well as being able to provide a very small active porous area (for example, ≤1 mm^2^ via electron beam lithography and maximum 4 cm^2^ using other lithography techniques). This has been hampering the membrane utilization in some important biomedical applications or lowering the possibility to extend the fabrication to the industrial scale. In summary, we described here a new methodology to fabricate polymeric isoporous membranes with pore density 10-fold higher than track-etched membranes with similar pore size, large active filtration area, with demonstrated preparation for 38.5 cm^2^, extendable up to 80 cm^2^ with the same equipment, using a combination of photolithography and dry reactive ion etching techniques. The fabrication methodology is environmentally friendly as it does not require solvents, and it is a short time production process, once the mask is obtained. The mask can be used innumerous times for the membrane production. By using preformed extruded Mylar dense films as starting materials, we eliminate the preparation of membranes by solution casting or solution spin-coating. For further reducing the pore size from a micro- to submicro-scale, a CVD process was applied. Finally, the membranes were successfully used for sorting plant organelles. They can be potentially applied to the fractionation of other biological systems. The Mylar porous membranes are transparent with a thickness of 2.5 µm, which could facilitate the visualization of the cell culture and cell imaging by white light microscopy. Moreover, the membranes are flexible and easy to handle and Mylar has excellent chemical and thermal resistance, allowing to extend the application to separations in non-aqueous solvents and harsher conditions. Furthermore, we have proved that the developed fabrication protocol can be easily adapted to other polymers, by demonstrating the successful fabrication of Kapton isoporous membrane (with similar pores sizes), which can be also customized to a larger variety of organic materials, including poly(vinylidene fluoride), polyacrylonitrile, polyethylene, polypropylene, crosslinked polyepoxides and many more.

## Methods

### Isoporous membrane fabrication

The isoporous membranes for biological separations were prepared using 2.5-μm thick Mylar (Chemplex Industries, INC.) and 7.5-μm Kapton (Dupont) as starting materials, submitted to a multi-step photolithographic/etching process, as depicted in Fig. 1.

#### Photomask fabrication

A pattern constituted by densely distributed 2-μm cylindrical pores and identical 2-μm interpore space was designed using the Tanner EDA L-Edit software. The total pattern area was delimitated by a 3-inches diameter, hence containing around 250 million pores. A μPG501 (Heidelberg Instruments) direct-writing tool printed the pattern on a triple layered photoresist-chromium-glass Photomask (5 × 5-inch from Nanofilm), during 3 hours, after 3 hours of pattern file format-conversion. After the exposure, the photomask was immersed in a developer (AZ-726 MIF) for 60 seconds to dissolve the photoresist on the exposed area. Then, the developer was thoroughly washed out with a deionized (DI) water spray for 30 seconds. After washing, the photomask was placed in a chromium etchant (Cr01 UN 3264, TechniEtch) bath at room temperature and the bath was gently agitated for 90 seconds. The photomask was then washed again with DI water and dried by blowing nitrogen. At the last step, the photomask was placed in an UV-LED system (UV-KUB 2 tool, KLOE) for 3 minutes to fixate the pattern. Once completed, this photomask could be further repeatedly used for the isoporous membrane fabrication. Analogous masks were designed for cylindrical pores with diameters 5, 10, 25 and 50 μm.

#### Tape-polyester film preparation

A 2.5-μm thick Mylar film (or 7.5-μm Kapton film) with 3-inches diameter was firstly attached to a special tape-film (Load Point, micro-machining solutions) coated with an acrylic adhesive layer. The tape is UV-sensitive and this provides an easy way of detaching after the pore fabrication process.

#### Photolithography

On the top of a silicon wafer (4-inch diameter from University Wafer), we applied an AZ-9260 photoresist, by spin-coating. The tape-polyester film was then accurately rolled on the photoresist-coated wafer, avoiding the formation of air bubbles. The photoresist functioned as a glue. The resulting laminate was then spin-coated with a 1.6 μm layer of another photoresist (AZ-5214). After that, the system was baked for 30 seconds at 110 °C. The baked laminate was exposed to UV light under the designed patterned photomask, using an EVG-620 nano-imprint photolithography tool. The UV light exposure run under a 80 mJ/cm^2^ dose and vacuum + hard contact mode. After the exposure, the system was immersed in a developing bath (AZ-726 MIF Developer) for 90 seconds, rinsed in DI water for 1 minute and dried by blowing nitrogen.

#### Dry-etching process

The pore fabrication was finalized with a dry-etching process, using an inductive-coupled plasma reactive ion etching (ICP-RIE) equipment (Oxford Instruments), which operated with the following parameters: sulfur hexafluoride (SF_6_) flow of 10 standard-cubic centimeters per minute (sccm) and oxygen (O_2_) flow of 30 sccm, pressure of 10 mTorr, radio-frequency power of 50 W, ICP power of 800 W. The etching process for Mylar was performed in 3 steps of 4 minutes-etching and 3 rest-breaks of 1 minute each. A longer etching time (total of 45 min) was applied for the Kapton film. The etched laminate was then washed with acetone for 1 minute, rinsed with isopropyl alcohol and DI water. After drying by blowing nitrogen, a final curing step was done with UV using a U-200 (POWATEC) system to release the attached tape from the finally obtained isoporous membrane.

### Submicron pore size reduction via chemical vapor deposition (CVD)

Selected membranes, previously prepared by photolithography and dry-etching with pore size 2 μm, were submitted to a partial poly-(chloro-p-xylylene) (Parylene C) deposition process, carried out using a Lab coater (Model PDS 2010) CVD equipment. The thickness of the uniform Parylene coating was controlled by the amount of the solid-phase dimer (Parylene powder) that was loaded into the system. Hence, in order to further reduce the pore size from 2 μm down to 0.7 μm we used 1 g of parylene powder.

#### Sample preparation

1 g of solid dichloro-[2,2]-para-xylylene (Parylene C dimer) was placed inside the evaporator chamber. The microporous membrane was fixed on top of a glass slide and then placed in a deposition chamber.

#### Deposition process

The CVD process involved three steps. First, the dimer was evaporated at 175 °C in the evaporator chamber. Then, the vaporized dimer entered the pyrolysis chamber to form chloro-p-xylylene monomeric gas at the 690 °C. Finally, when entering the deposition chamber, it condensed and polymerized as poly-(chloro-p-xylylene) on the microporous membrane at room temperature, reducing the pore sizes.

### Arabidopsis growth

The seeds were sterilized, by incubating them in a 1/4/3 (v/v/v) bleach/ethanol/water solution during 10 min, followed by four times washing with sterile DI water. The seeds were then stratified to synchronize their germination, by placing them at 4 °C for 48 h. The seeds were sown on Jiffy-7 pots to prevent contaminations from garden soil for four weeks at 23 °C, 60% relative humidity with an 16 h light/8 h dark long daily photoperiod in an environmentally controlled growth cabinet (Percival). Fertilizer (2 g/L) was added on the second and fourth weeks during the growth period. Preparation of Arabidopsis organelles homogenate. Four weeks old Arabidopsis plants were harvested (about 60 g of fresh tissue weight) and 300 ml of the extraction buffer (400 mм sucrose, 50 mм Tris-HCl pH 7.5), EDTA (3 mм), 0.1% Bovine serum albumin, dithiothreitol (2 mм) (DTT) (Sigma-Aldrich Chemicals), cOmplete™ protease inhibitor cocktail (Roche), and phenyl-methylsulfonyl fluoride (1 mм) (PMSF) (Sigma-Aldrich) were mixed in a Waring® blender. The tissue was homogenized three to four times with pulses of two seconds each. The extract was filtered through four layers of Miracloth to remove cell debris and unbroken cells. The filtrate was then centrifuged at 1,100 g for 5 minutes and the obtained pellet was discarded. The supernatant was further centrifuged at 18,000 g for 20 minutes. The supernatant was discarded and the pellet was gently suspended in a wash buffer (300 mм sucrose, 50 mм Tris-HCl pH 7.5, 3 mм EDTA, 0.1% Bovine serum albumin, 2 mм DTT (Sigma-Aldrich chemicals)), cOmplete™ protease inhibitor cocktail (Roche) and PMSF (1 mм) (Sigma-Aldrich). The last centrifugation step was repeated and the finally obtained pellet was suspended in wash buffer and used as the plant organelle homogenate for the fractionation experiments with the here manufactured membranes.

### Fractionation experiments

The plant organelle homogenate was successively passed through membranes of various pore sizes ranging from 50 µm, 25 µm, 10 µm, 5 µm and 2 µm by gravity flow without the application of any pressure, using Millipore Amicon 8010 cell with an active membrane area of 4.1 cm^2^. A cascade of cells with decreasing pore size, as shown in Fig. S4 was then used for the fractionation. The organelles that permeated through each membrane were properly fixated and imaged by scanning electron microscopy (SEM) on a Quanta 600 (FEI) microscope. The samples were mounted onto stubs and coated with a 3 nm layer of iridium. From the SEM images obtained from each fraction, organelles of various sizes were counted using the ImageJ analysis software.

## Supplementary information

Supplementary information.
