# Supplementary material for: Nanofabrication of Isoporous Membranes for Cell Fractionation
Source: Sci Rep. 2020 Apr 9;10:6138. doi: 10.1038/s41598-020-62937-5 (PMC7145805; doi:10.1038/s41598-020-62937-5)
Supplement: Supplementary file 1 — Supplementary information. [file 41598_2020_62937_MOESM1_ESM.docx]

Supplementary Information

**Nanofabrication of Isoporous Membranes for Cell Fractionation**

Ainur Sabirova^1^, Florencio Pisig Jr^2^, Naganand Rayapuram^3^, Heribert Hirt^3^,

Suzana P. Nunes^1^

^1^King Abdullah University of Science and Technology (KAUST), Biological and Environmental Science and Engineering (BESE) Division, Advanced Membranes and Porous Materials Center, 23955-6900 Thuwal, Saudi Arabia

^2^King Abdullah University of Science and Technology (KAUST), Nanofabrication Core Laboratory, 23955-6900 Thuwal, Saudi Arabia

^3^King Abdullah University of Science and Technology (KAUST), Biological and Environmental Science and Engineering (BESE) Division, Center for Desert Agriculture, 23955-6900 Thuwal, Saudi Arabia

**
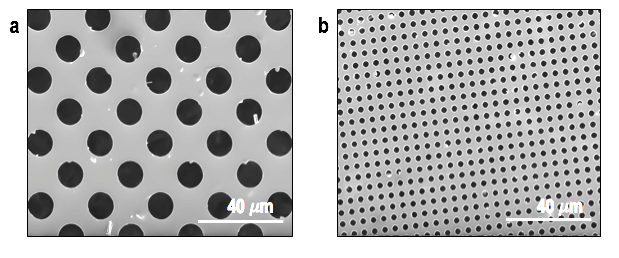
Figure S1. SEM images of Kapton membranes prepared by photolithography/dry reactive ion etching.** (a) 10 µm and (b) 2 µm pore sizes.


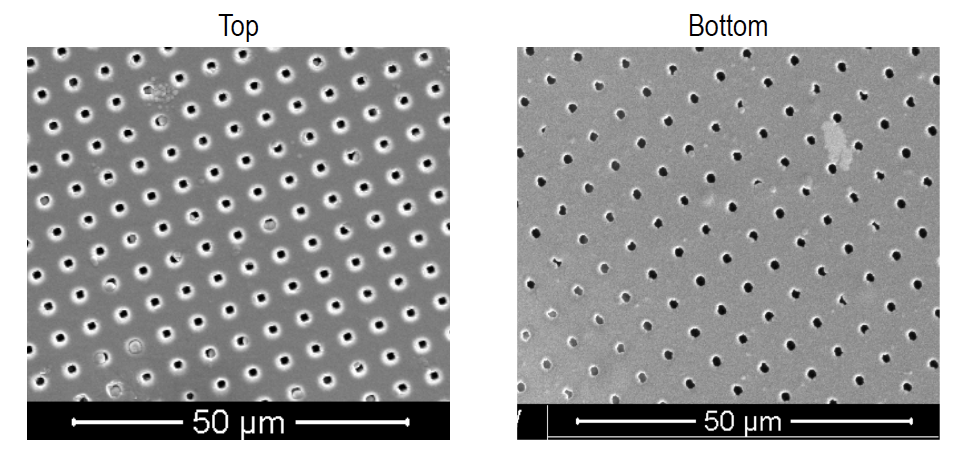


**Figure S2. SEM images of 2 µm Mylar membranes partially coated with Parylene by CVD.** Images of the top and bottom.


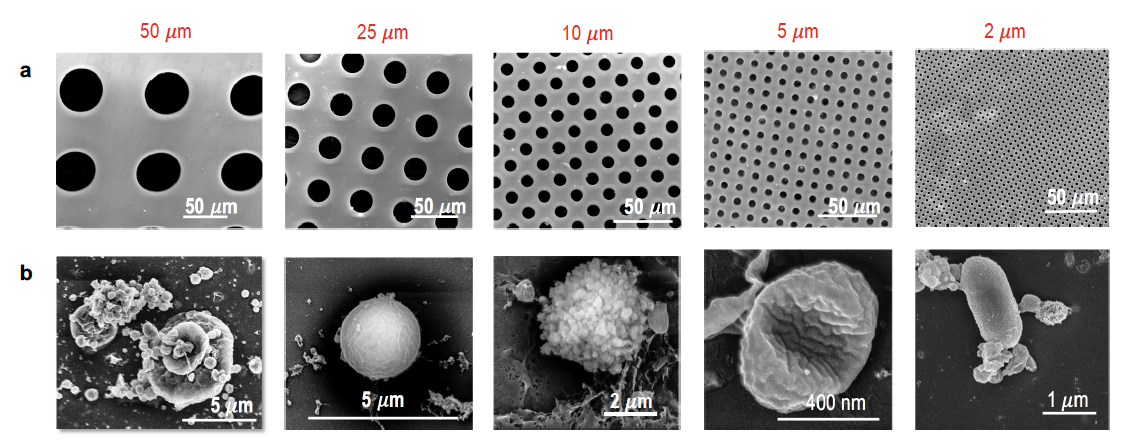


**Figure S3. Organelles separation by membrane filtration.** (a) SEM images of Mylar isoporous membranes fabricated with pore sizes of 2, 5, 10, 25 and 50 μm. (b) Details of organelles of different sizes permeated and fractioned through the membranes imaged above (complementary to Figure 4).


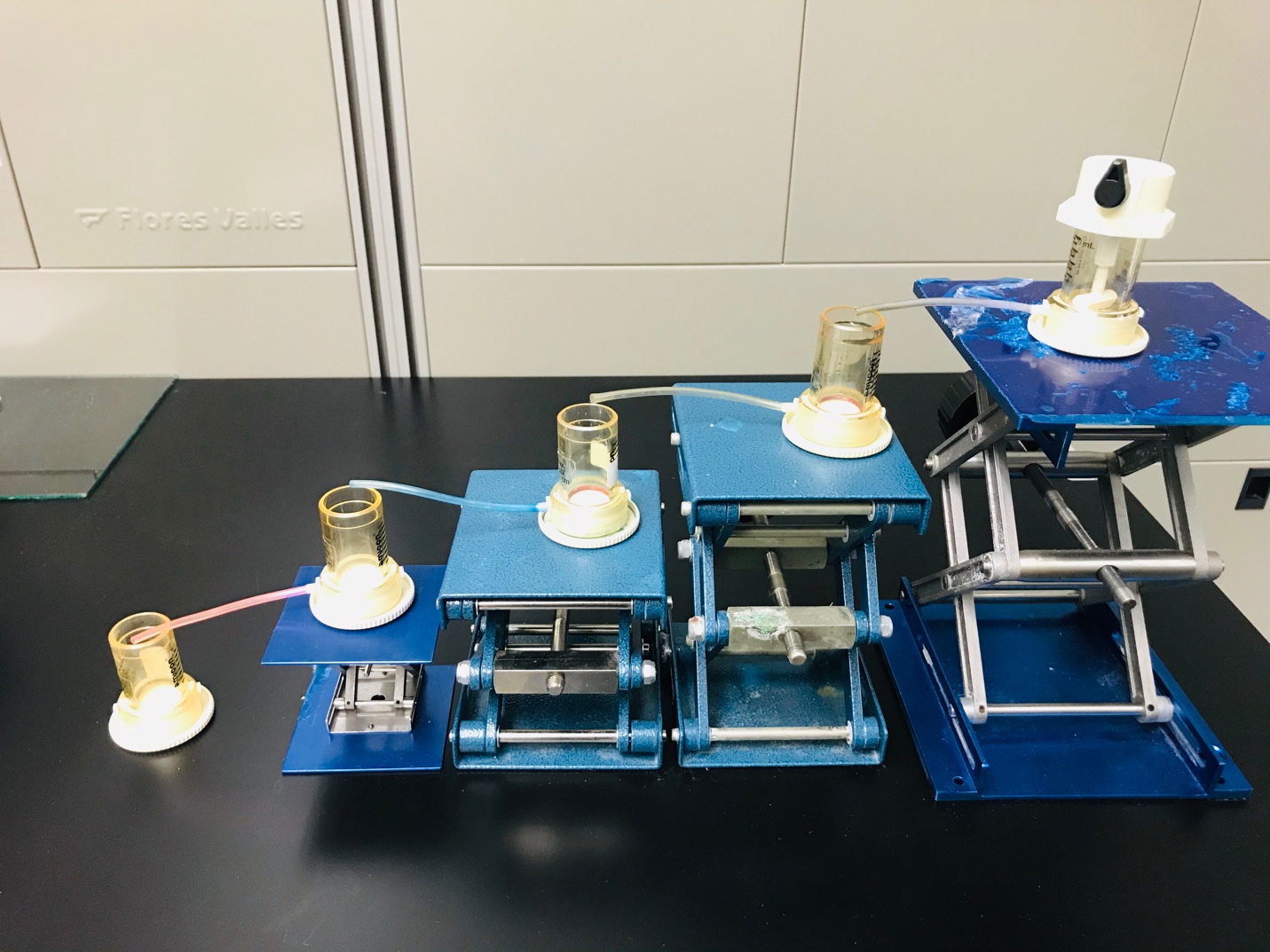


**2 μm**

**5 μm**

**10 μm**

**25 μm**

**50 μm**

**Figure S4.** Photograph of the experimental set-up for the plant organelles fractionation (size selective sorting).


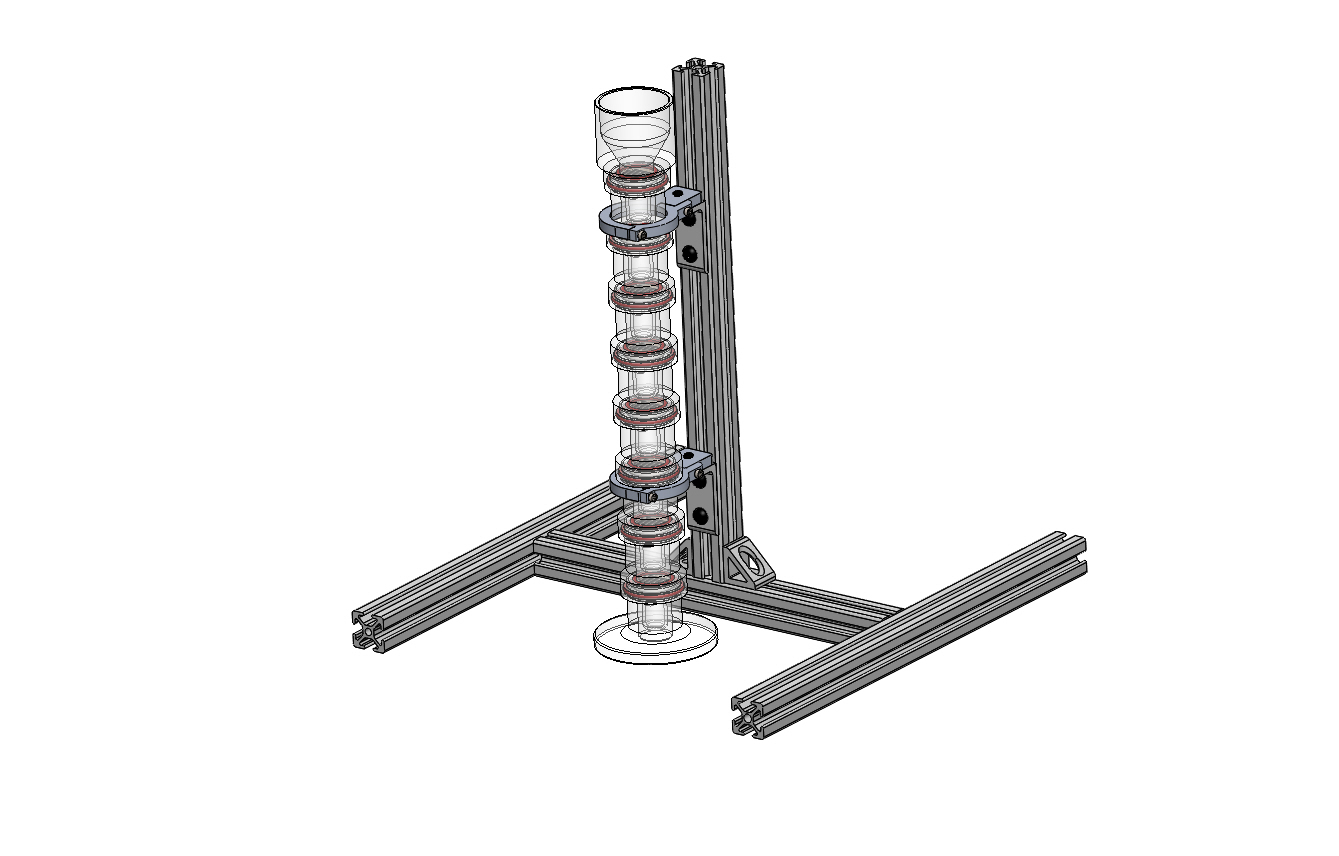


**Figure S5.** AutoCad design of a filtration set-up constituted by 8 single consecutive membrane cells.
